# Characteristics and outcomes of unplanned extubation in adult ICU: two-year retrospective observational cohort study

**DOI:** 10.62838/jccm-2026-0059

**Published:** 2026-10-30

**Authors:** Khalid Abdullah Alreyes, Alzahra Abdulwahed Alobaid, Ahmed Yahya Aljoni, Nada Hadi Saleem, Abdullah Hamed Alosaimi, Atwah Mohammed Alotaibi, Mutab Falah Alotaibi, Saad Majed Alyami, Nasser Abdulrahman Bin Jawan, Thamer Awadhallah Almutairi, Waleed Abdullah Althurwi, Naif Abdulrahman Aldawsari, Mohammed Saleh Alfaifi, Turki Faisal Alharbi, Aqeel Hamad Alali, Princess Rhea Julkarnain, Arul Prasath Lakshmanan, Waleed Th. Aletreby

**Affiliations:** Respiratory Care Administration, King Saud Medical City, Riyadh, Saudi Arabia.; Critical care Department, King Saud Medical City, Riyadh, Saudi Arabia.

**Keywords:** unplanned extubation, self extubation, accidental extubation.

## Abstract

**Objectives:**

Description of unplanned extubation rate, and characteristics of patients who experienced it.

**Methods:**

A two-year retrospective descriptive study conducted in a 120-bed closed ICU in Riyadh, Saudi Arabia. Adult mechanically ventilated patients who experienced UE were included. Data on demographics, airway management, staffing ratios, and clinical outcomes were analyzed. Results: Out of 4,054 mechanically ventilated patients, 226 experienced UE, to an incidence rate of 0.52 per 100 ventilator days. Majority of events were self-extubations (77.4%) and occurred during the day shift (61.5%). Median (IQR) distance from the endotracheal tube (ETT) tip to carina was 6.2 (4.6 – 7.8) cm, indicating relatively superficial positioning. Re-intubation was required in 57.5% of cases within 48 hours. Overall 28-day mortality rate was 22.6%. Accidental extubation patients were more frequently reintubated (90% vs. 48%) with a higher 28-day mortality rate (44% vs. 16.6%). Subgroups of younger age (≤39 years; RR 0.35), occurrence during the day shift (RR 0.56), successful avoidance of re-intubation (RR 5.54 if reintubation required), and self extubation (RR 2.66 if accidental) were associated with lower mortality risk.

**Conclusion:**

While the incidence of UE was relatively low, the high rate of self-extubation during peak activity hours (day shifts) and the superficial positioning of ETTs suggest a need for enhanced tube fixation and vigilance during sedation holidays. Younger age, day shift occurrence, avoiding the need for re-intubation, and self-extubation coincided with a lower observed risk of 28-day mortality. These findings are hypothesis-generating and highlight distinct clinical patterns that warrant further adjusted, prospective investigation.

## Introduction

Unplanned extubation (UE) is the unintended removal of an endotracheal tube (ETT) before it is planned by the treating team. It may occur as self-extubation, when the patient intentionally removes the ETT, or as an accidental extubation during routine care or procedures [1]. UE remains one of the most common adverse events in mechanically ventilated patients and continues to be an important quality-of-care indicator in the intensive care unit (ICU), given its potential association with respiratory compromise, emergency reintubation, prolonged hospitalization, and increased healthcare costs [2,3,4]. Although reported incidence varies widely across settings, UE remains clinically significant because of its implications for both patient safety and resource utilization [3].

The occurrence of UE is typically multifactorial. Patient factors like agitation, and inadequate sedation increase self-extubation risk, while mechanical factors, such as poor fixation, superficial positioning increase risk of accidental extubation [5,6,9]. In addition, staffing patterns, workload, and reduced surveillance may further increase vulnerability to UE [2, 3, 10, 11].

Uncertainties remain regarding the incidence and consequences of UE. Prior studies have reported inconsistent findings, likely reflecting variation in patient case mix, sedation practices, staffing models, and ICU organization across institutions [3, 4].

Accordingly, the present two-year descriptive study examined the incidence of UE in our institute, its characteristics and outcomes among mechanically ventilated adult ICU patients between January 2024 and December 2025, as this was not explored before, in one of the largest ICUs in the country. Specifically, we evaluated the demographic and clinical characteristics of UE events, including staffing ratios, patient demographics, ICU type, ETT position and fixation, duration of ventilation, and 28-day outcomes. We also calculated the incidence of UE per 100 ventilator days to allow comparison with previously published literature [12].

## Metods

### Design and setting

This was a descriptive study, conducted in the ICU of a large tertiary referral Ministry of Health hospital, in the central region of Saudi Arabia, and a member of Riyadh first health cluster. The ICU includes 120 beds, divided into several units, namely: Medical, Surgical, Respiratory, Trauma, Neuro-critical care, Burn, and Maternity units. It is a closed ICU, operated round the clock by intensivists, with a one to one nurse to Patient ratio. All ICU beds are fully equipped for invasive and non-invasive monitoring, and mechanical ventilation.

The study was approved by the local institutional review board (Approval number: H1RI-25-Feb26-03), with waiver of consent in view of its retrospective nature, and lack of intervention. However, the study fully complies with principles of protection of research participants, as outlined by the Declaration of Helsinki, and Saudi National Committee of Bioethics (NCBE).

### Operational definitions

Unplanned extubation: Any premature, unexpected, or uncontrolled removal of ETT from the patient’s trachea while receiving mechanical ventilation, occurring without a written physician’s order or a planned clinical weaning protocol. This includes both accidental and self extubation.

Self-extubation: An unplanned extubation where the patient is the direct agent of the tube removal. This occurs when a patient deliberately or inadvertently pulls, dislodges, or shakes out their own ETT due to agitation, delirium, or lack of sedation, using their hands, teeth, or vigorous head movement.

Accidental extibation: An unplanned extubation caused by external forces or during clinical manipulation, without any purposeful or non-purposeful physical intervention by the patient to pull the tube out.

Ventilation days: The cumulative number of calendar days a patient receives mechanical ventilation via ETT for any portion of that day.

28 day mortality: All-cause death occurring within exactly 28 days (672 hours) starting at ICU admission, not the event of UE.

### Inclusion and exclusion criteria

The study included adult patients (at least 18 years old), who were admitted to the ICU, and mechanically ventilated via an ETT (whether upon ICU admission, or during the ICU stay). Patients who experienced UE during their stay in the ICU, and had at least one chest X-ray performed within 24 hours prior to the unplanned extubation event. We included only UE in the key ICU admission, while excluding subsequent events whether in the same ICU admission or in a second admission, in order to maintain independency of the data.

### Exclusion Criteria

We excluded patients if they were not intubated during their ICU stay, did not experience UE, or had missing data with regards to: mortality, chest radiograph within the previous 24 hours, reason of UE, or re-intubation.

### Study objectives

The primary objectives were descriptive calculation of the unplanned extubation rate / 100 mechanical ventilation (MV) days, and to summarize the average ETT depth from the carina (cm) in the most recent chest x-ray prior to UE.

Secondary objectives included description of demographic and clinical characteristics of patients who had UE, including:

- Age
- Sex distribution
- Reason of ICU admission and intubation.
- Duration of mechanical ventilation.
- ICU shift (day / night) of occurrence of unplanned extubation.
- Incidence of re-intubation within 48 hours.
- 28-day mortality.
- Additionally, we compared self extubation to accidental extubation patients with regards to the same demographic and clinical characteristics, as an additional exploratory analysis.

We also included tertiary exploratory comparison of 28-day mortality between (A priori sub-groups):

- ICU shifts (Day / Night). The Day shift begins at 8:00 and is 12 hours long, and the Night shift begins at 20:00 till the next day.
- Reintubation or not.
- Males versus females.
- Median age of the cohort.
- Median distance from carina in chest x-ray, as measured by the assigned respiratory therapist.
- Accidental or self-extubation

### Data management

Eligible patients were identified through the daily flow sheets maintained by respiratory therapists, and additional relevant information were retrospectively extracted from the patients’ electronic health records. To ensure data accuracy, all entries were double-checked by a second reviewer. The collected data were recorded in a standardized Excel sheet and securely stored by the primary investigator on a password-protected computer.

It is a routine practice in our ICU for the assigned respiratory therapist to review the early morning chest radiograph – which is routinely done for all mechanically ventilated patients – and to measure the distance in centimeters of the tip of ETT to carina, similarly, the assigned respiratory therapists measure the cuff ’s pressure of ETT using hand held manometers (VBM Medizintechnik ® Germany) at the beginning of each of the two shifts of their 24 hours schedule, they also routinely assess the status of ETT fixation as either “Tight” or “Loose”, an ETT tie is considered loose if two fingers can be easily passed between the tie and the patient’s cheek. The assigned respiratory therapists document and report their measurements during the morning round.

### Statistical method

#### Sample Size

We planned to include all eligible patients within the study period, however, we estimated the minimum required sample size to estimate the mean distance of the ETT tip from the carina with a predefined precision. Based on prior literature, the standard deviation of this distance was assumed to be 1.5 cm [13]. To achieve a 95% confidence interval with a margin of error of 0.2 cm, the required sample size was calculated to be 217 patients, to ensure a precise estimate of ETT positioning.

#### Statistical Analysis

All data were analyzed using freely available software (R Studio) [14]. For descriptive outcomes, we summarized continuous variables using the mean and standard deviation (SD) if normally distributed, or the median and interquartile range (IQR) if distributional assumptions were not met, but both measures were presented for the average distance of ETT tip from carina. We assessed normality using Shapiro-Wilk test. Categorical variables were summarized using frequencies and percentages. We compared continuous variables (between self and accidental extubation) using student t-test or Mann Whitney U test as appropriate, while categorical variables were compared using Pearson’s Chi square test.

For the tertiary outcomes, comparisons between subgroups with regards to 28-day mortality were presented as relative risk (RR). Two – tailed tests were used, assuming statistical significance if p value < 0.05, and results were presented with corresponding 95% confidence intervals (CI).

All analyses are exploratory unadjusted, hence, the results should not be considered conclusive, and should be interpreted cautiously.

### Results

#### Incidence and Patient Flow

During the two-year study period (January 2024 to December 2025), a total of 7,239 patients were admitted to the ICU. Of these, 4,054 patients were mechanically ventilated, contributing a total of 43,060 ventilation days. UE occurred in 226 instances representing individual patients, without exclusions due to missing data (Figure 1), resulting in an overall incidence rate of 0.52 per 100 MV days (95% CI: 0.46 – 0.6).

**Figure 1:**
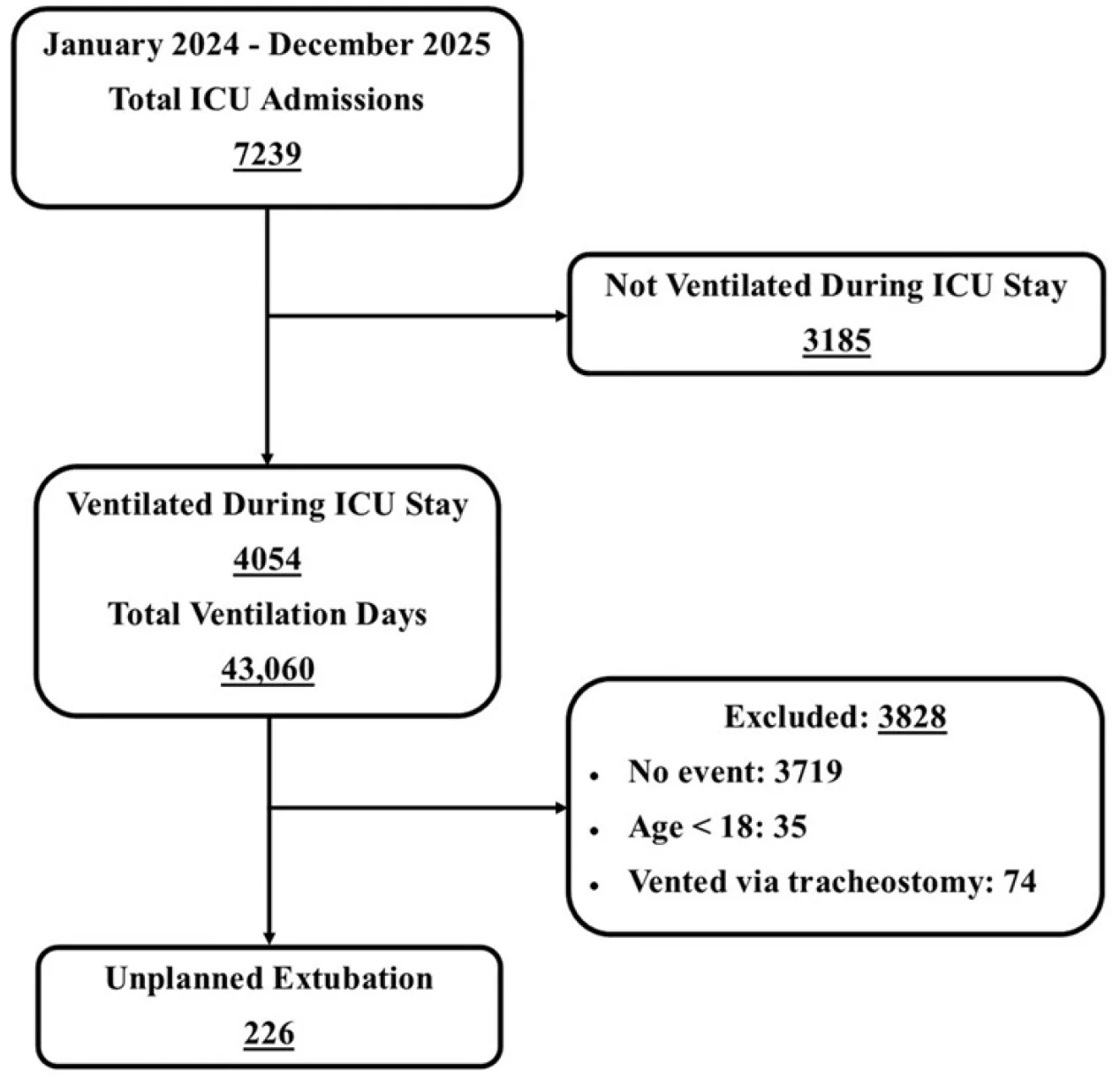
Flow diagram of patients’ enrollment

#### Demographic and Clinical Characteristics

The demographic profile and clinical characteristics of the cohort are summarized in Table 1. The majority of patients were males (n=182, 80.5%), with a median (IQR) age of 39 years (IQR: 27–54). The median duration of mechanical ventilation prior to the UE event was 3 days (IQR: 1–6).

The most frequent diagnostic category for ICU admission was poly-trauma (20.4%; Figure 2), while airway protection was the leading indication for endotracheal intubation (41.6%; Figure 3). Regarding the circumstances of the event, self-extubation was the primary cause, accounting for 77.4% (n=175) of all cases.

**Table 1:** Demographic and clinical characteristics of patients with unplanned extubation:

**Variable**	**Count (%) / Median (IQR)**
Gender	
Male	182 (80.5%)
Female	44 (19.5%)
Age (years)	39 (27 – 54)
Shift	
Day	139 (61.5%)
Night	87 (38.5%)
Number of patients simultaneously assigned to Respiratory Therapist	6 (6 – 7)
ETT Level (above carina) (Cm)	6.2 (4.6 – 7.8)
ETT Size (ID in mm)	7.5 (7 – 7.5)
MV days till incidence (Day)	3 (1 – 6)
Re-intubated within 48 hours	
No	96 (42.5%)
Yes	130 (57.5%)
Cuff pressure (cm H_2_O)	31 (26 – 38)
ETT Tie condition	
Tight	176 (77.9%)
Loose	50 (22.1%)
Possible Cause	
Accidental	50 (22.1%)
Self-extubation	175 (77.4%)
Unclear	1 (0.4%)
28 Day Mortality	
Alive	175 (77.4%)
Died	51 (22.6%)

ETT = endotracheal tube, ID = internal diameter, cm = centimeter, IQR = inter-quartile range

**Figure 2:**
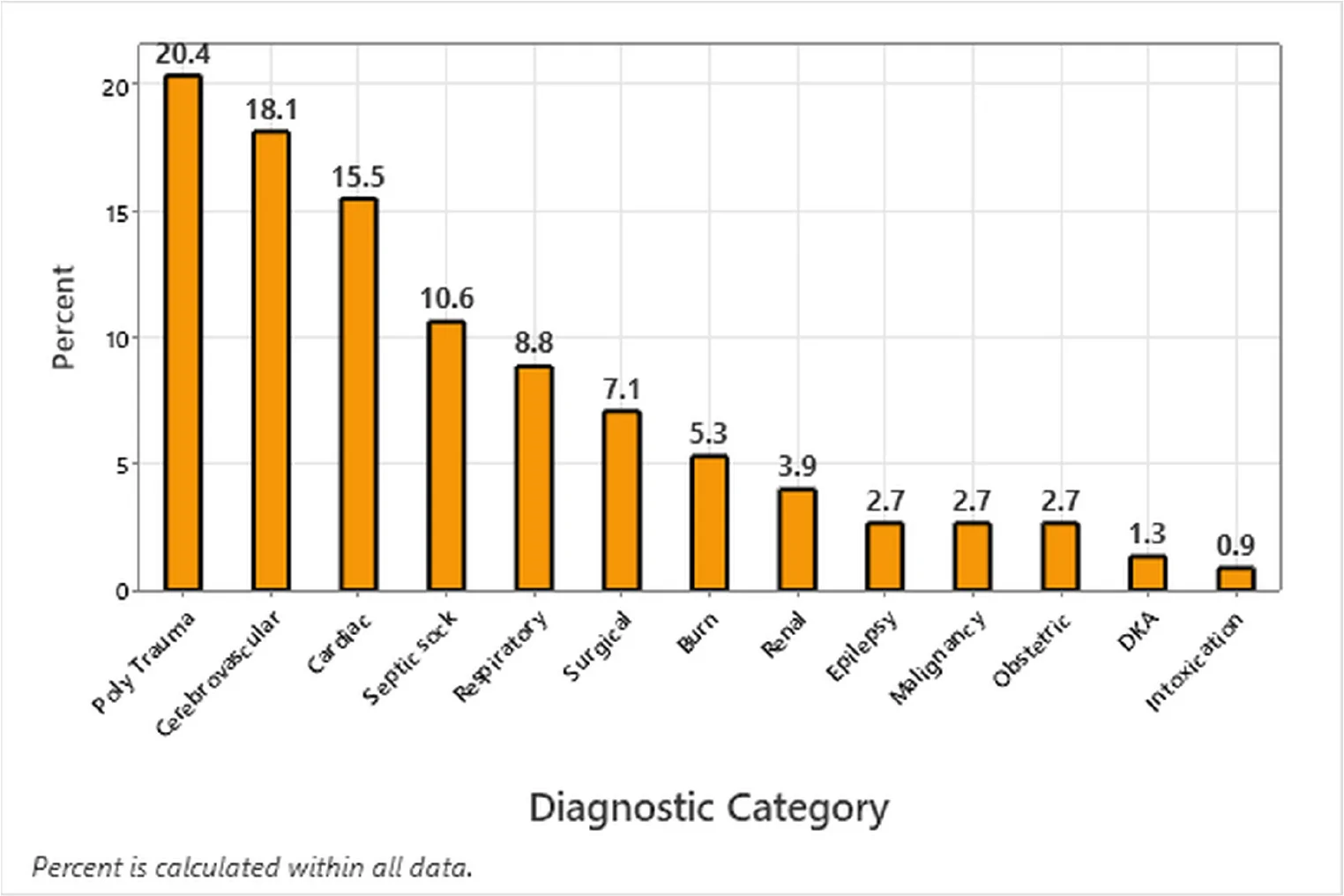
Diagnostic categories of patients with unplanned extubation DKA = diabetic keto-acidosis.

**Figure 3:**
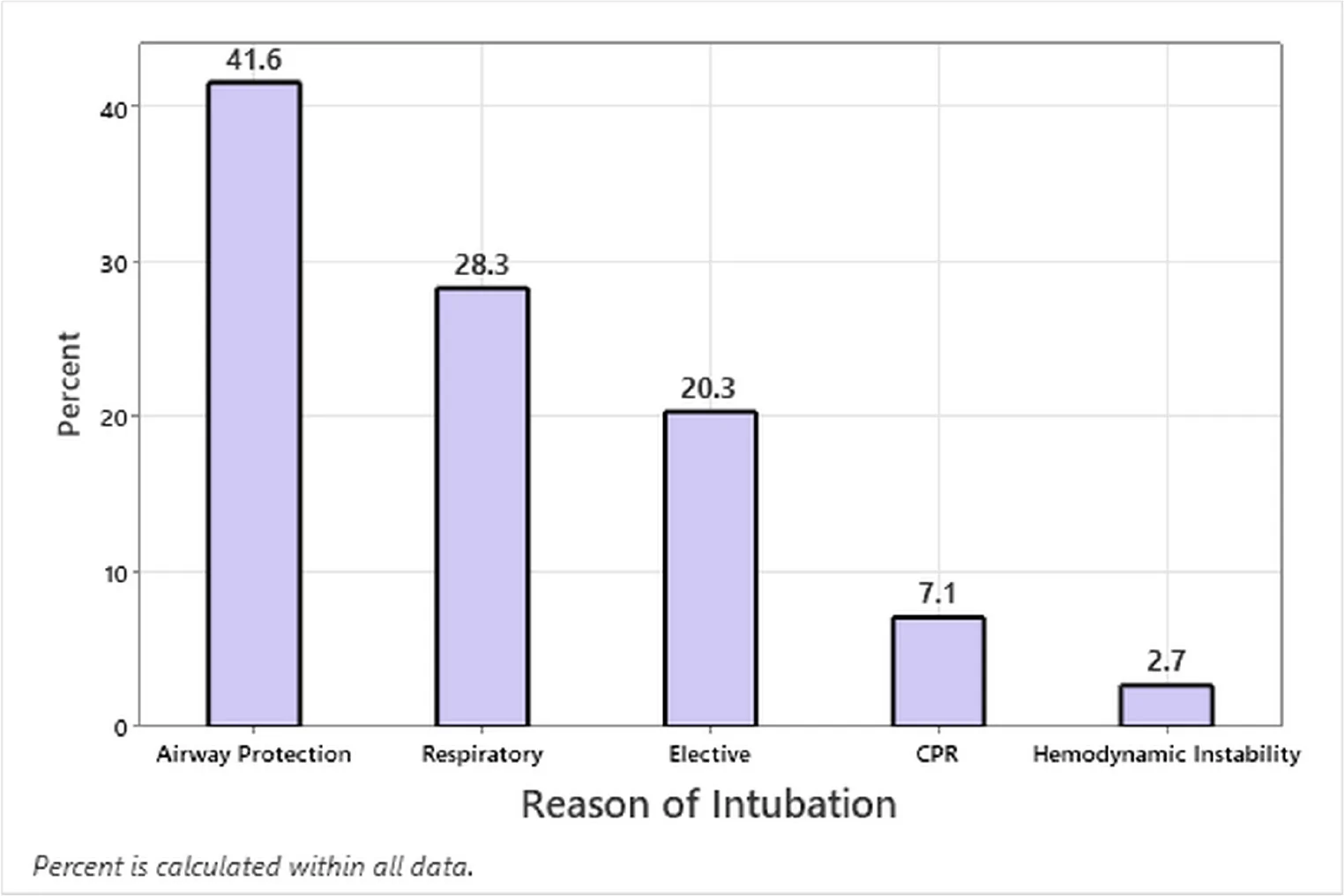
Indications of intubation CPR = cardiopulmonary resuscitation.

#### Airway Management and Staffing

Analysis of ETT positioning via the last chest X-ray prior to UE revealed a median distance from the carina of 6.2 cm (IQR: 4.6 – 7.8), corresponding to a mean (SD) of 6.3 (2.2) cm (data were normally distributed). The median cuff pressure was 31 cmH2O (IQR: 26–38). The majority of ETTs were considered adequately secured (n=176, 77.9%).

In terms of organizational factors, more than half of the UE events occurred during the day shift (n=139, 61.5%). At the time of the event, the median number of patients assigned to each respiratory therapist was 6 (IQR: 6–7).

#### Post-Event Outcomes and Mortality

Following UE, 57.5% of patients (n=130) required re-intubation within 48 hours. The overall 28-day mortality rate for the cohort was 22.6% (n=51, 95% CI: 17.3% – 28.6%).

Exploratory comparisons between accidental and self-extubation (Table 2) indicate that accidental extubation patients were more frequently reintubated within 48 hrs. (90% versus 48%), percent difference (95% CI) = 42% (31% to 53.1%; p < 0.001), they also had a higher 28-day mortality (44% versus 16.6%), percent difference (95% CI) = 27.4% (12.6% to 42.2%; p < 0.001). Keeping in mind that the lack of key variables (such as patient status at the time of UE, sedation regimen, RASS score, delirium, use of restraints, spontaneous breathing trials, sedation interruption, and ventilatory settings) may have confounded the results.

**Table 2:** Comparisons between Self extubation and Accidental extubation sub-groups:

**Variable**	**Accidental (n=50) Mean (SD) / (n, %)**	**Self extubation (n=175) Mean (SD) / (n, %)**	**Mean / Percentage difference (95% CI; p value)**
Age (years)	37.9 (17.6)	42.3 (17)	−4.4 (−10 to 1.2; 0.1*)
Sex (Males)	40 (80%)	141 (80.6%)	−0.6% (−13.1% to 12%; 0.9)
Shift (Night)	24 (48%)	62 (35.4%)	12.6% (−3% to 28.1%; 0.1)
ETT tip above carina (cm)	6.7 (2.7)	6.2 (2)	0.5 (−0.3 to 1.3; 0.1*)
MV days	4.7 (5.2)	4.2 (4.7)	0.5 (−1.1 to 2.1; 0.9*)
Cuff pressure (cmH2O)	31.4 (7.8)	32.3 (9.8)	−0.9 (−3.6 to 1.7; 0.9*)
ETT fixation (Loose)	9 (18%)	40 (22.9%)	−4.9% (−17.2% to 7.5%; 0.4)
Reintubation within 48 hrs.	45 (90%)	84 (48%)	42% (31% to 53.1%, < 0.001)
28-day Mortality	22 (44%)	29 (16.6%)	27.4% (12.6% to 42.2%; < 0.001)

One patient is excluded for unknown extubation category

* Mann Whitney U test

ETT = endotracheal tube, cm = centimeter, SD = standard deviation, CI = confidence interval.

Unadjusted exploratory subgroup analysis for 28-day mortality (Figure 4) shows that patients at or below the median age (≤39 years) had a lower observed mortality compared to older patients (RR 0.35, 95% CI: 0.20 – 0.62). Similarly, events occurring during the day shift were associated with a lower observed mortality (RR 0.56, 95% CI: 0.34 – 0.90). The need for re-intubation within 48 hours was associated with an increased observed mortality (RR 5.54, 95% CI: 2.46 – 12.45). While accidental extubation had a higher risk of mortality compared to self extubation (RR 2.66, 95% CI: 1.68 – 4.19). No significant differences in mortality were observed based on sex, ETT distance from the carina, or respiratory therapist-to-patient ratios (p > 0.05).

**Figure 4:**
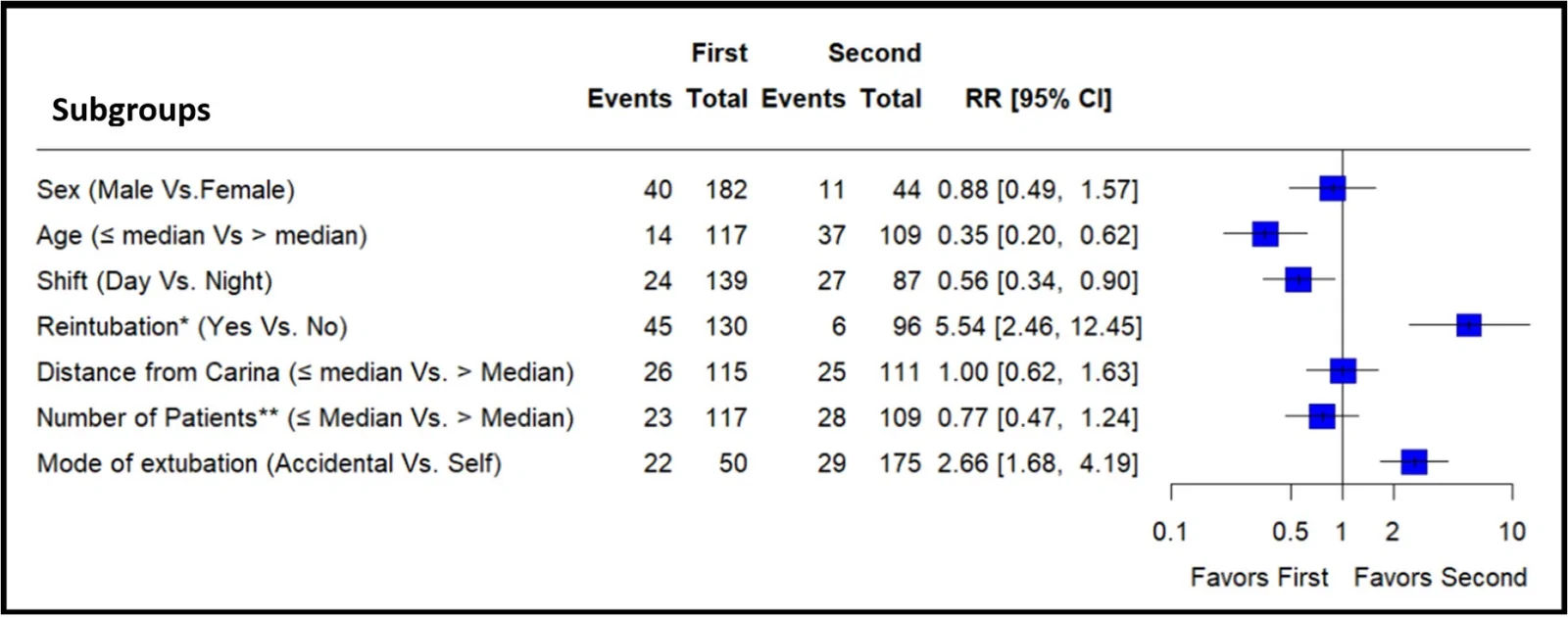
28-day mortality subgroups analysis *Reintubation within 48 hours **Number of patients simultaneously assigned to respiratory therapist

## Discussion

In this study, the incidence of UE was 0.52 / 100 MV days (95% CI: 0.46 – 0.6). Most of events occurred for males, during the day shift, with an average distance of ETT tip from carina of 6.2 cm, and a cuff pressure of 31 cmH2O. The majority of events were due to self extubation, and most of the patients were reintubated within 48 hours. Overall 28-day mortality was 22.6%. Younger age, occurrence during the day shift, self extubation, and when reintubation was not constituted within 48 hours had lower mortality. However, these findings should be interpreted as hypothesis-generating rather than evidence of independent prognostic factors. Incidence of UE widely varies, it is reported to be 0.3 to 4.2 / 100 mv days [4], and in some reports, as low as 0.1 / 100 mv days [3], to as high as 6.6 / 100 mv days [15]. This seems to reflect variations in practice, and differences between ICUs at the staffing, workload intensity, and operational levels. Our ICU, with a 1:1 nursing ratio, and 1:6 - on average - respiratory therapist to patient, is performing relatively better than others who reported higher rates [12, 16].

Interestingly, most of UE events occurred during the day shift, a finding that is echoed by others [1, 4]. The day shift is when most of interventions and procedures take place, whether at the bedside or outside the ICU requiring mobilization. Hence, it is understandable that adverse events occur during that time, although our study didn’t assess such a link. Furthermore, that was not the case always, Chang et al [17] reported a majority of UE occurring during night shifts, and they associated that with lower nursing experience or education.

Coupled with frequent occurrences during the day shifts, our study as well as others observed a majority of self extubations, rather than accidental [1, 4, 12, 17]. This relation seems to point to spontaneous breathing trials, and assessment of readiness of extubation with sedation vacation, which usually take place in the morning, and may be accompanied by patients’ agitation, followed by self extubation [18], However, we should not overlook the possibility of under-sedation in an attempt to reach the recommended light sedation [19], on the expense of more frequent adverse events.

Not surprising, the majority of patients in our study required re-intubation within 48 hours, as was observed by others [1, 12, 17], reaching up to 88% in a systematic review [3]. This seems logic, since the extubation was not planned, nor was the readiness for extubation assessed. Guillemin et al [1] indicated that significantly more accidental UE end up re-intubated as compared to self-extubation, which also appears to be logic, since the self-extubation occurs mostly during sedation vacation or a spontaneous breathing trial to assess the patients’ readiness, which may result in a successful extubation, this was the case in our study as well.

The average distance between the tip and the carina in our cohort seems to be just above or within the upper limit of acceptable distance range of 2 – 6 cm [20], or 5 ± 2 cm [21]. A median (IQR) distance of 6.2 (4.6 – 7.8) is relatively superficial (although we can’t be sure due to the lack of comparison to a control group) and may be the result of frequent positioning of the head, either during procedures or routine care, this is supported by a similar case control study [22] that reported a median (IQR) distance of 5.2 (3.8 – 6.5) cm, while earlier measurements were 4.9 (4 – 5.4). A slightly above recommendation cuff pressure of 20 – 30 cmH2O [23] was observed in our cohort, which may indicate that an adequate cuff pressure does not guarantee tube stability, nor does it assure against UE, and the same can be said about tightly fitted ETT fixation.

The subgroup analysis of 28-day mortality identified a lower risk if there was no reintubation, since the detrimental impact of reintubation are well documented [24]. Lower age had a lower risk of mortality, probably due to their higher resilience and tolerability. While the lower mortality risk of UE occurring during the day shift may be attributed to higher staffing level and vigilance. Lower risk of mortality for self-extubations may be due to the readiness of the patients to planned extubation, as shown by their lower re-intubation rate. However, those apparently plausible explanations remain speculative, as such conditions were not systematically recorded in our study. Counter to our expectations, there was no difference in mortality risk between high and low patient assignment to the respiratory therapist, although with a lean to lower mortality with fewer assigned patients, however, this didn’t reach the level of significance.

We believe that this study provides some insights about the incidence and associated characteristics of UE, which should be reflected on clinicians’ practice in the form of adequate patient monitoring, and sufficient staffing particularly during periods of interrupted sedation or transfer, constant checking and verification of ETT position, and extra attention to elderly patients to avoid UE and subsequently reintubation. Further investigations are needed that include a group without UE, and adequately powered comparative studies that differentiates between self and accidental extubation with regards to their outcomes.

## Limitations

Our study is subject to numerous limitations. It is a single center study, reflecting practice in our hospital, and its results may not be generalized to other institutes. It is a retrospective observational study harboring all the inherent limitations and biases of such designs. We did not compare to a “control” group without UE. Finally, the comparison between self and accidental extubation, and the subgroup analysis were underpowered, and their results should be cautiously interpreted, furthermore, the results may have been confounded by several variables that were not systematically recorded and reported in our study, such as the absence of illness-severity measures and data on sedation and delirium, potential selection and classification biases, and the unadjusted nature of the exploratory analyses making it difficult to interpret because these groups may represent clinically distinct populations.

## Conclusion

The incidence of UE in our ICU is relatively low, and is common among males, during the day shift. The ETT are relatively superficial, although over inflated. Most of the events were due to self-extubation, with an overall 28-day mortality rate of 22.6%. Younger age, occurrence during the day shift, self extubation, and the successful avoidance of re-intubation coincided with lower 28-day mortality rates. Given the unadjusted nature of this descriptive study, these factors should be interpreted as prognostic markers of lower risk rather than definitive survival advantages.

## References

[1] Guillemin J, Rieu B, Huet O, Villeret L, Pons S, Bignon A (2024). Prospective multi-center evaluation of the incidence of unplanned extubation and its outcomes in French intensive care units. The Safe-ICU study. Anaesth Crit Care Pain Med..

[2] Bouza C, Garcia E, Diaz M, Segovia E, Rodriguez I (2007). Unplanned extubation in orally intubated medical patients in the intensive care unit: a prospective cohort study. Heart Lung..

[3] da Silva PS, Fonseca MC (2012). Unplanned endotracheal extubations in the intensive care unit: Systematic review, critical appraisal, and evidence-based recommendations. Anesth Analg..

[4] Kiekkas P, Aretha D, Panteli E, Baltopoulos GI, Filos KS (2013). Unplanned extubation in critically ill adults: Clinical review. Nurs Crit Care..

[5] Ju TR, Wang E, Castaneda C, Rathod A, Abe O (2022). Superficial placement of endotracheal tubes associated with unplanned extubation: A case-control study. J Crit Care.

[6] Kumar AA (2024). Endotracheal Cuff-pressure Monitoring in ICU: A Standard of Care Yet to be Standardized, and Often Neglected. Indian J Crit Care Med..

[7] Lucchini A, Bambi S, Galazzi A, Elli S, Negrini C, Vaccino S, Triantafillidis S, Biancardi A, Cozzari M, Fumagalli R, Foti G (2018). Unplanned extubations in general intensive care unit: A nine-year retrospective analysis. Acta Biomed.

[8] Minda Z, Samuel H, Aweke S, Mekete G, Seid A, Eshetie D (2022). Magnitude and associated factors of unplanned extubation in intensive care unit: A multi-center prospective observational study. Ann Med Surg (Lond).

[9] Cosentino C, Fama M, Foà C, Bromuri G, Giannini S, Saraceno M, Spagnoletta A, Tenkue M, Trevisi E, Sarli L (2017). Unplanned Extubations in Intensive Care Unit: evidences for risk factors. A literature review. Acta Biomed.

[10] Ali K, Almahdi M, Algarni SS, Alsaif S, Alharbi RO, Alqahtani MA, Aldubaian R, Alsharif M, Castro M, Esclanda A, Althubaiti M, Alrahili M, Alshareef M, Homedi A, Ali I (2025). Trends, risk factors, and outcomes of unplanned extubation in a neonatal intensive care unit: a seven-year retrospective study. Front Pediatr.

[11] Mion LC, Minnick AF, Leipzig R, Catrambone CD, Johnson ME (2007). Patient-initiated device removal in intensive care units: a national prevalence study. Crit Care Med..

[12] Li P, Sun Z, Xu J (2022). Unplanned extubation among critically ill adults: A systematic review and meta-analysis. Intensive Crit Care Nurs.

[13] Varshney M, Sharma K, Kumar R, Varshney PG (2011). Appropriate depth of placement of oral endotracheal tube and its possible determinants in Indian adult patients. Indian J Anaesth..

[14] R Core Team (2022). R: A language and environment for statistical computing.

[15] Liu K, Liu Z, Li LQ, Zhang M, Deng XX, Zhu H (2022). Predictive value of the unplanned extubation risk assessment scale in hospitalized patients with tubes. World J Clin Cases..

[16] Aladin M, Buckley L, Maloney M, Rojanaporn P, Gombar T, Lewis A (2025). Unplanned extubation prevention in the neuroscience ICU at a High Reliability Organization. J Clin Neurosci.

[17] Chang LC, Liu PF, Huang YL, Yang SS, Chang WY (2011). Risk factors associated with unplanned endotracheal self-extubation of hospitalized intubated patients: a 3-year retrospective case-control study. Appl Nurs Res.

[18] Schweickert WD, Gehlbach BK, Pohlman AS, Hall JB, Kress JP (2004). Daily interruption of sedative infusions and complications of critical illness in mechanically ventilated patients. Crit Care Med..

[19] Devlin JW, Skrobik Y, Gélinas C, Needham DM, Slooter AJC, Pandharipande PP (2018). Clinical Practice Guidelines for the Prevention and Management of Pain, Agitation/Sedation, Delirium, Immobility, and Sleep Disruption in Adult Patients in the ICU. Crit Care Med.

[20] Harris RJ, Baginski SG, Bronstein Y, Kim S, Lohr J, Towey S, Velichkovich Z, Kabachenko T, Driscoll I, Baker B (2021). Measurement of Endotracheal Tube Positioning on Chest X-Ray Using Object Detection. J Digit Imaging..

[21] Shetty A Evaluation of endotracheal tube position. Reference article, Radiopaedia.org.

[22] Gueret RM, Tulaimat A, Morales-Estrella JL (2020). Self-Extubation Revisited: A Case-Control Study. Respir Care..

[23] Sadovyi V, Kuchyn I, Bielka K, Horoshko V, Sazhyn D, Sokolova L (2024). Endotracheal tube cuff pressure assessment: expectations versus reality. Anaesthesiol Intensive Ther..

[24] Dadam MM, Pereira AB, Cardoso MR, Carnin TC, Westphal GA (2024). Effect of Reintubation Within 48 Hours on Mortality in Critically Ill Patients After Planned Extubation. Respir Care..

